# Phenolic Compound Profile by UPLC-MS/MS and Encapsulation with Chitosan of *Spondias mombin* L. Fruit Peel Extract from Cerrado Hotspot—Brazil

**DOI:** 10.3390/molecules27082382

**Published:** 2022-04-07

**Authors:** Giovanna Oliveira de Brito, Bruna Cabral Reis, Eduardo A. Ferreira, Nilton T. Vilela Junqueira, Lívia C. L. Sá-Barreto, Fulvio Mattivi, Urska Vrhovsek, Eliana Fortes Gris

**Affiliations:** 1 Graduate Program in Health Sciences and Technologies, Faculty of Ceilandia, University of Brasilia, Centro Metropolitano, Conjunto A, Lote 01, Brasilia 72220-275, Brazil; giovanna.odebrito@gmail.com (G.O.d.B.); brunacabralreis@gmail.com (B.C.R.); 2 Faculty of Ceilandia, University of Brasilia, Centro Metropolitano, Conjunto A, Lote 01, Brasilia 72220-275, Brazil; eduardoantonioferreira@gmail.com or; 3 Brazilian Agricultural Research Corporation—EMBRAPA, Embrapa Cerrados, Rodovia BR-020, Km 18, Planaltina 73310-970, Brazil; nilton.junqueira@embrapa.br (N.T.V.J.); liviabarretofarm@hotmail.com (L.C.L.S.-B.); 4 Department of Cellular, Computational and Integrative Biology—CIBIO, University of Trento, Via E. Mach 1, 38098 San Michele all’Adige, Italy; fulvio.mattivi@unitn.it; 5 Edmund Mach Foundation, Via E. Mach 1, 38010 San Michele all’Adige, Italy; urska.vrhovsek@fmach.it

**Keywords:** taperebá, cajá, bioactive compounds, UPLC-MS/MS, microencapsulation, chitosan, spray drying

## Abstract

Taperebá (*Spondias mombin* L.) is a native species of the Brazilian Cerrado that has shown important characteristics such as a significant phenolic compound content and biological activities. The present study aimed to characterize the phenolic compound profile and antioxidant activity in taperebá peel extract, as well as microencapsulating the extract with chitosan and evaluating the stability of the microparticles. The evaluation of the profile of phenolic compounds was carried out by UPLC-MS/MS. The in vitro antioxidant activity was evaluated by DPPH and ABTS methods. The microparticles were obtained by spray drying and were submitted to a stability study under different temperatures. In general, the results showed a significant content of polyphenols and antioxidant activity. The results of UPLC-MS/MS demonstrated a significant content of polyphenols in taperebá peel, highlighting the high content of ellagic acid and quercetin compounds. There was significant retention of phenolic compounds when microencapsulated, demonstrating high retention at all evaluated temperatures. This study is the first to microencapsulate the extract of taperebá peel, in addition to identifying and quantifying some compounds in this fruit.

## 1. Introduction

The Cerrado biome has an extensive variety of endemic species, presenting a range of exotic fruits that have peculiar and intense sensory characteristics, attributes that arouse the interest of the food industry [1]. In addition, bioactive substances found in these fruits provide environmental protection and health benefits [2]. The adverse conditions found in the Cerrado, such as frequent fires, seasonal rains, and nutrient-poor soil, are partially responsible for the phytochemical properties of plants and can influence the profile and quantity of these substances [3]. Although this biome has very rich biodiversity and includes the largest tropical savanna in South America, there are many endangered species due to land use conversion and the scarcity of areas of environmental preservation. It is considered one of the 34 “hot spots” for conservation in the world [4], containing numerous plants and fruits with medicinal benefits that have not yet been scientifically explored [3].

The taperebá (*Spondias mombin* L.), also called cajá, cajá verdadeiro and cajá-mirim, is a member of the Anacardiaceae family and can be found in tropical areas of America, Asia and Africa, presenting as a small ovoid drupe with thin yellowish skin and a bittersweet taste [5]. This wild fruit from the Cerrado has shown important characteristics, due to the significant content of some phenolic compounds [6,7,8,9], demonstrating biological effects such as cytotoxic activity in ovarian cancer cells [9], gastroprotective action in rats [10] and antioxidant ability [8].

In addition to taperebá pulp, which is the part of the fruit used in most studies [5,8,10], its peel also has a considerable concentration of total polyphenols [7]. The taperebá peel can be considered industrial waste, and it is usually discarded, wasting its economic and functional potential [11]. Other studies also verified this potential in some exotic fruits, such as Gualberto et al. (2021) [12], in a study that evaluated bioactives in industrial waste from acerola (*Malpighia emarginata* L.) umbu (*Spondias tuberosa* L.), genipap (*Genipa americana* L.) and guava (*Psidium guajava* L.) and verified high phenolic content in all of them. Therefore, research involving waste, especially peel, can strengthen the development of value-added products, such as edible films, probiotics and nanoparticles, among others [13]. The use of industrial by-products reduces waste, converting them into beneficial economic results for society and the environment [14].

Bioactives, such as the polyphenols in taperebá [6,7,8,9], may present advantageous characteristics, such as anti-cancer, anti-inflammatory and antioxidant properties. Studies evidenced that the ingestion of these compounds can contribute to the reduction of oxidative damage and chronic diseases [15,16,17]. However, their high instability can represent a disadvantage, as is well-known, since they are sensitive to external stimuli when extracted from fruit [17,18,19,20,21], which can compromise their beneficial properties and limit possible applications in different areas [21,22,23,24,25]. For this reason, efforts have been made to develop and optimize different technologies for improving the stability of polyphenol compounds and then to obtain them with improved bioavailability, stability, and storage duration. Regarding these technologies, microencapsulation stands out [7,18,21,23,24,25,26].

Polymeric materials are generally used for the microencapsulation process. Chitosan, which is a biopolymer, has some advantages, such as low cost, low toxicity, biocompatibility and biodegradability, in addition to having already been used in the spray drying method [27]. This type of process, by spraying, is already widely used in the food industry to increase the shelf life of food [28], and it has also been used for the microencapsulation of compounds such as polyphenols extracted from tropical fruits from Brazil [26]. Its good utility is justified by characteristics such as high encapsulation efficiency, cost-effectiveness, applicability in industry and reproducibility [26,28].

Therefore, the objective of this research is to characterize the profile of phenolic compounds and antioxidant activity in taperebá peel extract, as well as microencapsulating, characterizing and evaluating the stability of chitosan microparticles containing taperebá peel extract under different temperature conditions.

## 2. Results

### 2.1. Quantification of Total Phenolics and Evaluation of the Antioxidant Activity of Taperebá Peel Extract

The quantified value of total polyphenols in taperebá peel was 557.65 ± 29.73 mg of gallic acid equivalents (GAE) per 100 g of peel.

The in vitro antioxidant activity measured from the capture of the DPPH radical by the ethanol extract of taperebá peel was 51.62 ± 1.27 µMTEAC/g of peel. The in vitro antioxidant activity measured from the ABTS radical capture was 98.57 ± 2.49 µMTEAC/g of peel.

### 2.2. Evaluation of the Profile of Phenolic Compounds in Taperebá Peel by UPLC-MS/MS

Through UPLC-MS/MS, several phenolic compounds were quantified, such as flavonols, phenylpropanoids, benzoic acid derivatives, coumarins, stilbenes, dihydrochalcones, flavones, and flavonones (Table 1). Among the flavonols, 14 compounds were quantified. Quercetin was the most abundant compound, followed by myricetin and Kaempferol-3-Glc. Interesting values were also verified for syringetin and isorhamnetin-3-Glc. Phenylpropanoids were quantified, such as cinnamic acid, as well as other derivatives of this compound, such as some hydroxycinnamic acids and sinapyl alcohol. Among these, chlorogenic acid was found in the greatest quantity, followed by *p*-coumaric and cinnamic acid. Ellagic acid was the benzoic acid derivative found in the greatest quantity in taperebá peel, followed by gallic acid. Among the quantified coumarins, esculin stands out. Among the stilbenes quantified in this experiment, *cis*-piceid stands out due to its high quantified values. Interesting contents of *trans*-piceid and *trans*-resveratrol were found. Sinensetin and Luteolin-7-*O*-Glc were the most prominent flavones. In addition, glycosylated hydroquinone arbutin was quantified.

### 2.3. Characterization of Chitosan Microparticles Containing Taperebá Peel Extract (TMP)

The yield obtained in the encapsulation of the ethanol extract of the taperebá peel with chitosan was 19.5% and the encapsulation efficiency was 55.4%, with the polyphenol content equal to 149.59 ± 4.3 mg GAE/g of the microparticle.

**Figure 1:**
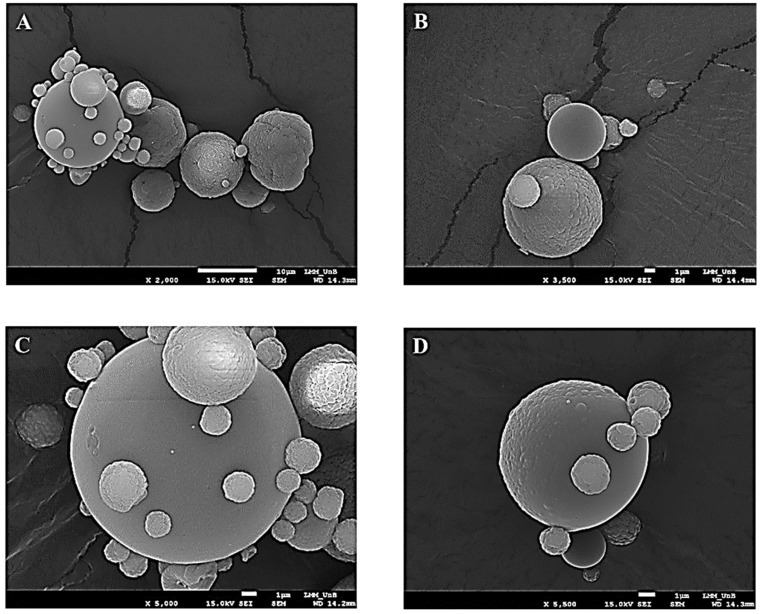
Photomicrograph obtained by scanning electron microscopy at magnifications of 2000 times (**A**), 3500 times (**B**), 5000 times (**C**) and 5500 times (**D**) for taperebá peel microparticles (TMPs) obtained by spray drying using chitosan as an encapsulating agent.

The analysis of the morphology of the TMPs formed was possible through photomicrographs generated by scanning electron microscopy (SEM). In Figure 1A, it can be seen that these presented the shape of spheres of varying sizes with a wrinkled or smooth surface, as shown in Figure 1B–D.

### 2.4. Stability Study of Taperebá Peel Microparticles (TMP) and Lyophilized Peel Extract (LPE)

**Figure 2:**
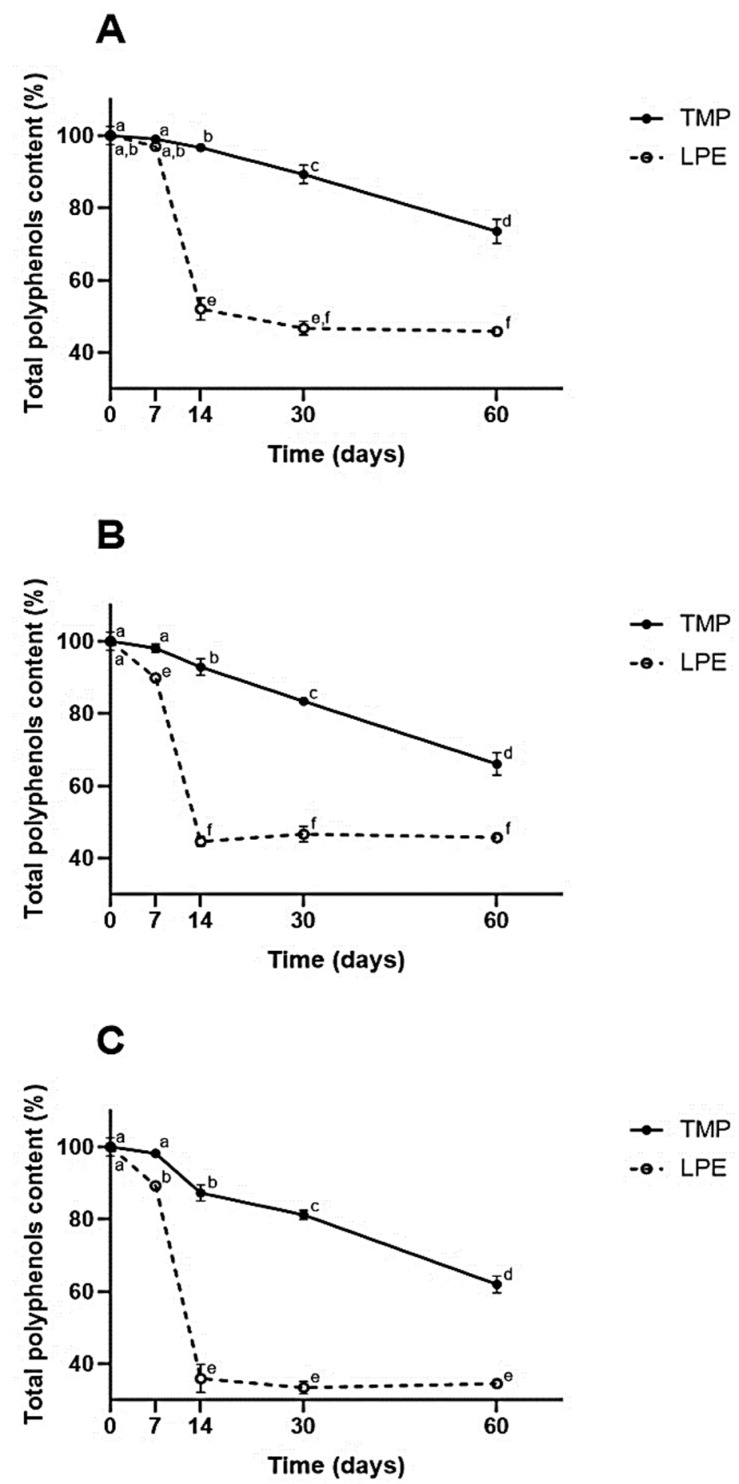
Total polyphenol content of samples submitted to different temperatures over 60 days. TMP = taperebá peel microparticles with chitosan; lyophilized peel extract (LPE). (**A**) Refrigerated temperature ~2–6 °C. (**B**) Room temperature ~25 °C. (**C**) Heat stress temperature ~40 °C. Different letters in each graph represent statistically significant differences (*p* ≤ 0.05) according to the Tukey HSD test.

The stability analyses of the microencapsulated and lyophilized extracts were carried out on the day of production, on the seventh day, on the fourteenth day, on the thirtieth and on the sixtieth day. The results of these analyses are shown in Figure 2.

As can be seen in Figure 2, the results demonstrate that, when the extract was microencapsulated, there was more significant retention of its phenolic compounds at all temperatures studied, during the 60 days of the experiment, when compared to LPE (*p* ≤ 0.05).

It can be seen that, from the seventh day, except for the refrigeration temperature (which happened on the fourteenth day), TMPs already demonstrated greater retention of phenolic compounds (*p* ≤ 0.05). At the end of the experiment, the TMPs obtained retention of ~75% at the refrigerated temperature and ~65% at the heat stress temperature.

At the end of the experiment, for the TMPs, there was the greatest retention when refrigerated (~75%). As for the EECs, it was observed that on the thirtieth day there was no significant difference between the refrigeration and ambient temperature (~45%) (*p* ≤ 0.05). Significant degradation of EECs was observed between the seventh and fourteenth days (*p* ≤ 0.05), with a loss of more than half of the phenolic values (less than 50%). In the TMPs, there was a slight decrease in retention during the first few days; a more significant decrease was observed between the thirtieth and sixtieth day of the experiment (*p* ≤ 0.05).

When observing the results from the point of view of different storage temperatures, it was found that the heat stress temperature was the one that most degraded the samples, as expected, demonstrating the great sensitivity of phenolic compounds to thermal stress. It was found that microencapsulation significantly protected the phenolics in the extract, with a retention of ~60%, while the LPE showed a retention of ~35% (*p* ≤ 0.05).

## 3. Discussion

### 3.1. Quantification of Total Phenolics and Evaluation of the Antioxidant Activity of Taperebá Peel Extract

Phenolic compounds are extremely important for the antioxidant potential in plants, as they are part of the defense mechanisms that plants use. To date, this is the most explored mechanism, as most phytochemicals studied have this important activity [2]. Thus, it is essential to quantify these compounds in fruits, as well as their antioxidant activity [29].

Some works have pointed out a high content of phenolic compounds in tropical fruits from the Cerrado biome, Brazil [7,26]. Among these fruits, taperebá stands out, showing a high content of total polyphenols both in the peel, as in the present study (557.65 ± 29.73 mg GAE/100 g), and in the pulp, as demonstrated by Bataglion et al. (2015) [9]. In this study, the authors verified that the dry pulp of taperebá presented a higher content of phenolic compounds (TP) (712.72 ± 8.71 μg/g) when compared to other fruits, such as açaí-do-Amazonas (264.53 ± 10.56 μg/g) (*Euterpe precatoria*). Aniceto et al. (2021) [8] also found a similar trend in the values of phenolic compounds from taperebá (~1300 mg GAE/100 g of commercial frozen pulp) when compared to murici (*Byrsonima crassifolia* (L.) Kunth, ~300 mg GAE/100 g of commercial frozen pulp). Another piece of research, carried out by Abiodun et al. (2020) [30], quantified interesting values of phenolic compounds in taperebá seeds (*Spondias mombin* L.) (~240.00 GAE/g seed).

Studies with the peel of other fruits from the Cerrado biome have also shown them to be rich in bioactives. For example, the peel of jabuticaba (*Plinia cauliflora*), native to this region, demonstrated a content of ~1100 mg GAE/100 g [26]. Araticum or marolo (*Annona crassiflora* Mart.) is an exotic fruit from the Brazilian Cerrado, and in a study carried out by Arruda et al. 2018 [31], its peel presented a total polyphenol content of 31.65 ± 0.32 mg GAE/g, which is higher than the value found in pulp (20.49 mg GAE/g dry weight) and seed (12.68 mg GAE/g dry weight). Cerrado cherry (*Eugenia calycina* Cambess) showed a high concentration of phenolic compounds (the sum of the contents of all phenolic compounds detected by LC/MS-MS) ~7000.0 µg/g (fresh weight) in the pulp and ~2000 µg/g (fresh weight) in the seeds [32]. These results reinforce the potential of this biome in the production of phytochemicals that can be beneficial to health. These characteristics may be consequences of exposure to high ultraviolet radiation and water stress [3], as the hypothesis is that the level of bioactives is higher where resources are scarcer.

Taperebá peel is considered a residue, and considering its bioactive potential, this is a type of material that can be used in several areas, such as food, pharmaceuticals and cosmetics. This possible use can provide a reduction in waste, generating positive results in the economic field and also for the environment [14].

Studies exploring materials that are considered waste, such as mango (Kensington Pride), grapefruit (Thompson) and lime (Tahitian) peel [33], as well as the study by Cabral et al. (2018) [26] with jabuticaba peel, emphasize the importance and support the transformation of these residues into by-products, enabling their use.

The applicability of this use has already been demonstrated in the literature. The extract of taperebá bagasse was considered a potent natural antioxidant that can be used to decrease protein degradation and lipid oxidation in foods. It is a sustainable and cheap alternative that guarantees the intelligent use of industrial waste, according to Santana Neto and co-workers (2021) [34]. Another example is the applicability of rambutan husks (*Nephelium lappaceum*), with 10% of their production being used for food purposes, as a polysaccharide gel [35].

Both DPPH and ABTS are commonly used as free radicals to assess the antioxidant activity of plant extracts. Hydrogen donation results in a discoloration of the solution, which is quantified to indicate its elimination potential [36]. The present study revealed an antioxidant capacity of 51.62 ± 1.27 µMTEAC/g of peel for the DPPH radical and 98.57 ± 2.49 µMTEAC/g of peel for the ABTS radical. Aniceto et al. (2021) [8] found an antioxidant potential against the ABTS radical of 188.24 µMTEAC/g, for frozen taperebá pulp (*Spondias mombin* L.), which was higher than the values found for murici (*Byrsonima crassifolia* (L.) Kunth) 79.49 µMTEAC/g. Another interesting study, with industrial by-products from another fruit of the Spondias genus, the umbu (*Spondias tuberosa* L.), found high antioxidant power (DPPH 2440.3 µMTEAC/100 g of dry weight) [12]. A survey that evaluated 12 different ecotypes of *Spondias purpurea* L. identified ABTS contents that ranged from ~8.23 mMTEAC/100 g to ~1.89 mMTEAC/100 g [37] in one of the kinds of fresh fruit. Another set of research results demonstrates the high antioxidant power both in taperebá and in other fruits of the Spondias genus.

The importance of this type of analysis is due to the pathophysiology that involves free radicals in the body, as in high concentrations they can damage several types of biomolecules, being the reason for or the consequence of many inflammatory diseases [29]. Despite this, the physiological role of these radicals is crucial during plant germination. What happens after harvesting is that morphological changes are no longer necessary, and for this reason antioxidant agents need to avoid the action of free radicals [38].

Thus, plants have several antioxidant mechanisms, including the production of phytochemicals that exert this action. Phenolic compounds have a phenolic ring that has the ability to stabilize and displace unpaired electrons, and this gives it an antioxidant capacity. For this reason, the assessment of antioxidant activity is strongly present in numerous scientific studies, as it is one of the most important biological activities in the body, such as cardiac remodeling after myocardial infarction [39]. It can also influence several other actions, such as: anti-cancer, anti-inflammatory mechanisms, cardioprotective, and antimicrobial, among others [29].

### 3.2. Profile of Phenolic Compounds by UPLC-MS/MS in Taperebá Peel

To our knowledge, and within the specific compounds quantified in this study, several bioactives were quantified in *Spondias mombin* peel for the first time: coumarins, monolignol, stilbenes, chalcones, flavones and flavonones, and arbutin. Regarding the quantification of compounds in *Spondias mombin*, in general, it is verified in the literature that studies with the leaves, and in some cases with the pulp of this fruit, are more commonly carried out.

Regarding flavonoid compounds, our results are very important, since it is already known in the literature that some compounds of this class have a therapeutic effect on inflammatory diseases, atherosclerosis, thrombosis, cerebral ischemia, diabetes, Alzheimer’s disease and pathogenic microbial diseases [40]. In addition, flavonoids have beneficial effects on the cardiovascular system, due to their ability to reduce the oxidation of low-density lipoproteins, thus improving the lipid profile and the ability to produce vasodilation and regulate apoptotic processes in the endothelium, also exerting antioxidant activity [41]. Quercetin, rutin, rhamnetin and kaempferol have already been identified in extracts from taperebá leaves as having antiangiogenic and antitumor activity [42]. Brito et al. (2018) [43] identified quercetin (0.017 mg/mL) in taperebá juice. Although the major flavonol in our study was quercetin, our results for rutin showed an overall concentration of this compound (1.326 ± 0.08 mg/g of peel). Hernández-Ruiz et al. (2018), in Mexico [44], verified the content of rutin in *Spondias* ssp. in the edible part of this fruit (0.053 mg/g). Comparing our results with other exotic fruits, we found interesting values for the peel and pulp of araticum (*Annona crassiflora* Mart.), in which the authors quantified rutin (8.30 µg/g in the peel and 9.31 µg/g in the pulp) and quercetin (~8.30 µg/g in the peel and 21.83 µg/g in the pulp) [31]. The results found in the present study for the quantification of compounds in the peel seem to be much higher, despite the differences in the studied parts of the plant, as well as the expression of the results.

Among the quantified phenylpropanoids, the highest values were found for chlorogenic acid (10.236 ± 0.211µg/g), followed by *p*-coumaric acid (1.868 ± 0.211 µg/g). Cabral et al. (2016) [45] also found high levels of chlorogenic acid in *Spondias mombin* leaves, as did Silva et al. (2020) [46]. Furthermore, these last authors also verified anti-inflammatory activity from this leaf extract in human neutrophils. A study with some exotic fruits identified chlorogenic acid from methanolic extracts elaborated by shaker extraction in genipap (*Genipa americana* L.) (16.35 µg/g) and umbu (*Spondias tuberosa* L.) (20.84 µg/g) [12]. These values vary according to the fruit and are in line with our result. Gualberto et al. (2021) [12] also quantified *p*-coumaric acid in these fruits and obtained values for the methanolic extracts prepared by shaker extraction, which ranged from ~10 to ~60 µg/g, values higher than that found in taperebá peel. Arruda et al. (2018) [31] quantified bioactive compounds from araticum (*Annona crassiflora* Mart.), an endemic fruit of the Cerrado. In its peels, the authors quantified chlorogenic acid and *p*-coumaric acid in free, esterified and glycosylated forms. Chlorogenic acid was only found in its free form (13.24 ± 0.39 μg/g), while the *p*-coumaric acid compound was quantified in greater amounts in its esterified form (4.46 ± 0.05 μg/g), followed by its free form (1.37 ± 0.06 μg/g) and glycosylated (0.31 ± 0.02 μg/g). Among these results, the quantification of sinapyl alcohol stands out, a monolignol, cinnamic acid derivative, precursor of some coumarins and stilbenes, which was quantified for the first time in *Spondias mombin*. This compound is known to be complete in the biosynthesis of lignin, which is the main structural component of the cell walls of higher land plants [47].

Ellagic acid was the benzoic acid derivative found in the highest concentration in taperebá peel, followed by gallic acid. These values found are substantially significant. Ellagic acid is presented, in some studies with *Spondias mombin* leaves, as a species marker, in addition to acting as a gastric protector [43,45]. Brito et al. (2018) [43] found values of 0.069 mg of ellagic acid/mL of extract of taperebá leaves, cultivated in Paraíba, Brazil, and Cabral et al. (2016) [45] reported 19.4 mg/g of leaf extract cultivated in Rio Grande do Norte, Brazil. Our results for the peel of this fruit are significantly higher than those reported by these authors in the leaves and point to a higher content of this compound in this part of the plant. This finding is very interesting since ellagic acid has a proven antioxidant capacity [48]. Gallic acid is also considered a chemical marker for leaf extract, being useful for the quality control of products with *Spondias mombin* [49]. In a study with the edible part of the *Spondias* ssp, 0.040 g of gallic acid/g of extract was found [44] and in the leaves of *Spondias mombin* a content of 0.101 mg/mL [43]. For the first time, to our knowledge, benzoic acid derivatives, namely methyl gallate and syringaldehyde, were quantified in *Spondias mombin* peel.

Esculin, the main coumarin identified in this study (4.016 ± 0.371 µg/g), is classified as a simple coumarin, and this group has biological activities that include antitumor, anti-HIV, antibacterial, anti-inflammatory, anticoagulant and antioxidant [50].

Stilbenes are compounds with relevant biological effects already described in the literature, such as anti-inflammatory, antioxidant, hypolipidemic and anti-cancer activities [51]. Values quantified in this experiment showed high levels of *cis*-piceid (~20 µg/g), as well as *trans*-piceid (~3.5 µg/g). *Trans*-resveratrol was quantified, in lower concentrations (~0.7 µg/g). Cis-piceid and trans-piceid were found and quantified in a study with different types of wines, with 19.71 mg/L and 2.19 mg/L, respectively, in which the amount of cis-piceid was considerably higher, as in the present study [52]. *Trans*-resveratrol was quantified in a study with hydroalcoholic extraction of different industrial plant waste products, in which it was identified only in grape marc (*Vitis vinifera* L.) (0.07 mg/L) and grape stalks (*Vitis vinifera* L.) (2.36 mg/L) [53]. Such results demonstrate significant values for bioactive compounds in industrial by-products, which are wasted by the disposal of these materials.

Phloridzin was the dihydrochalcone found in the highest concentration. This compound is known to be partially responsible for the biological activity in apple trees (*Malus domestica* Borgh), being present in several parts of the plant, as demonstrated in the study by Táborský et al. 2021 [54], in the Opal variety (bark: 91.7 mg/g; leaves: 82.5 mg/g; twigs: 46.0 mg/g; flower buds: 53.9 mg/g). Furthermore, it is partially responsible for the biological activities attributed to apples (*Malus domestica*), being initially extracted from the root but also present in the peel and pulp. Phlorizin has great potential as a hypoglycemic agent, as it is a competitive inhibitor of sodium-glucose co-transporter 2 (SGLT2), responsible for approximately 90% of glucose reabsorption [55]. Many studies have demonstrated the beneficial health effects of phloridzin, such as anti-diabetic, anti-inflammatory, anti-hyperglycemic, anti-cancer, and antibacterial activities, as well as cardioprotective, neuroprotective, immunomodulatory and antioxidant action [56]. Among the flavones, in the present study, sinensetin presented the highest concentration (~1.5 µg/g), whereas the flavonone quantified was naringenin (~1.0 µg/g). Sinensetin, found abundantly in citrus peel, has shown anti-inflammatory actions in respiratory diseases, also demonstrating anti-cancer action, being a potential candidate in the treatment of T-cell leukemia [57]. Regarding naringenin, research that studied bioactive compounds in different fruits quantified this compound at between ~10 and ~1600 µg/g, depending on the fruits, type of extraction method and the extractor solvent [12], values higher than that found by us. Even with less elevated values, as shown by Gualberto et al. (2021) [12], our findings are interesting since naringenin can act in inflammatory disorders such as sepsis, fulminant hepatitis, fibrosis and cancer, and it can be used as an immunomodulator [58]. As far as we know, no studies quantified sinensetin, phloridzin, and esculin in *Spondias mombin* L.

To our knowledge, arbutin was detected in the genus Spondias for the first time. This hydroquinone is an interesting compound with proven antioxidant action due to its reducing power and is widely used in cosmetics as a skin depigmenter [59].

Despite the differences in the results expressed, our results demonstrate a high content of quantified phenolic compounds in the peel and possibly point to a higher content than in other parts of the plant. It is known that fruit peel can have a higher content of phenolic compounds compared to other parts of the fruit and that inedible parts of the plant are useful as a natural, highly potent and sustainable antioxidant [34].

### 3.3. Characterization of Chitosan Microparticles Containing Taperebá Extract (TMP)

In view of the high instability of phenolic compounds, and their consequent difficulty in applicability, the present study produced TMPs containing taperebá peel extract from medium molecular weight chitosan polymer by means of the spray drying method. These TMPs were viable and capable of encapsulating the extract.

The formation of TMPs and the morphology were evaluated using SEM (Figure 1A–C). The same structure pattern was found by Cabral et al. (2018) [26], who described that after the addition of the jabuticaba peel ethanol extract, the morphology of the chitosan microparticles with jabuticaba extract demonstrated a smoother surface—when compared to the microparticle without extract—indicating the interaction between the chitosan polymer and extract components, as in the present study.

In another work, microparticles containing phenolic extract of saffron petals (*Crocus sativus* L.), also formed by the same method, using maltodextrin as polymer, showed the same morphology pattern [60]. Another preparation demonstrated the same pattern of microparticle morphology, in which there was a complex coacervation between cationic polyelectrolyte chitosan and oppositely charged anionic surfactant, and then spraying with Vitamin E as content [61].

These findings demonstrate a pattern of microparticle formation by the spray drying method, which is an interesting method because it is simple, reproducible and presents low residual levels of toxic solvent, compared to other methods [27,62].

The photomicrographs of the present study demonstrate a non-cracking appearance of the TMPs, a characteristic that can give the product greater stability, as there is a reduction in the interaction with air [63]; although it can also interfere with the release of the gastrointestinal content [60].

The yield and encapsulation efficiency of the taperebá peel extract obtained was 19.5% and 55.4%, respectively. These are considered interesting results, since the phenolic compounds have thermal sensitivity, and the spray drying method is used at high temperatures, even for a very brief period. The encapsulation material used, chitosan, has the characteristic of forming a film when it dries on a smooth surface, which can reduce the process’ yield [27].

Studies with other types of materials to be encapsulated and using the same methodology, with chitosan, also showed yield results similar to ours. Minoxidil microencapsulation showed ~45% yield, on a pilot scale [27], while Cabral et al. (2018) [26] obtained a 40% yield with jabuticaba peel extract microencapsulation. Another well-known factor in the literature, and in practice, which influences the yield of this process, is the production of microparticles on a small scale since on an industrial scale the loss of material is considerably less significant.

Cabral et al. (2018) [26] performed encapsulation efficiency analyses also based on the evaluation of the content of phenolic compounds, as in the present study, and found a result of ~90%. This difference between the results can be explained by the fact that they are different species, with different values and composition of phenolic compounds. However, the results with *Spondias mombin* L. are also very promising.

### 3.4. Stability Study of Taperebá Microparticles (TMP) and Lyophilized Peel Extract (LPE)

As observed in the results presented, the microencapsulation with chitosan of taperebá peel extracts from the Cerrado promoted the protection of the phenolic compounds, at all storage temperatures, during the 60 days of the study. Similar protection has also been verified by other authors. Cabral et al. (2018) [26] reported significant retention values of phenolic compounds in jabuticaba peel extract microparticles (*Plinia cauliflora*) prepared with chitosan when compared to LPE. In that study, the authors reported retention of phenolic content in the microparticles of approximately 75% and 100%, after 30 days, in the heat stress temperature, and after 60 days under refrigeration, respectively.

Microencapsulation with other types of polymers and bioactive compounds also demonstrates the protection of these actives at different times and temperatures. Yingngam et al. (2018) [64] described the thermal stability of maltodextrin microparticles with the extract of *Antidesma puncticulatum* Miq., at 4 °C, 25 °C and 45 °C, where they reported retention values of ~90%, ~80% and ~50%, respectively, of the anthocyanin content on the thirtieth day of the study (*p* ≤ 0.05). Such results demonstrate, as is widely known, the sensitivity of phenolic compounds to high temperatures. Like Yingngam et al. (2018) [64] and Cabral et al. (2018) [26], we also believe that more studies need to be carried out with a longer storage period, as well as in relation to other variables that can contribute to the degradation of these compounds, such as interaction with air, incidence of light and humidity, among others. Another study evaluated chitosan as an encapsulating agent in the protection of the essential oils of lemongrass (*Cymbopogon flexuosus*), geranium (*Pelargonium* ssp.) and copaiba (*Copifera officinalis*) by atomization, as in our study. The results demonstrated the protective capacity of chitosan and that this polymer matrix can protect the encapsulated oils, providing greater thermal stability [65].

The use of microencapsulation technology for the protection of phenolic compounds becomes especially relevant when we consider their susceptibility to various external factors, as they are highly unstable substances [66]. Thus, this type of process can be used to slow down the degradation of these compounds, providing greater stability and thus greater application [18,26], as reported in the present study.

This increase in the stability of these compounds enables their use in different industrial areas. In the food industry, the demand for value-added foods has increased substantially, and for this reason, there is a lot of recent research on these processes. An example is the addition of concentrated grape juice microparticles to jelly recipes, which showed relevant phytochemical and antioxidant activity. These microparticles were elaborated from lyophilization using a whey-chitosan protein isolate system. Furthermore, the same work evaluated the in vitro gastric digestibility, which demonstrated a protective effect that allowed a slow release of anthocyanins [67].

## 4. Materials and Methods

### 4.1. Solvents and Reagents

Folin, ethanol, chitosan, methanol (LC-MS grade), acetonitrile (LC-MS grade), chloroform anhydrous stabilized with 0.5−1% ethanol and formic acid were purchased from Sigma-Aldrich (St. Louis, MO, USA). Most of the chemical standards are commercially available and were obtained from different suppliers (Supplementary Materials Table S1). The hydroxycinnamoyltartaric acids (*trans*-caftaric acid, *trans*coutaric acid, and *trans*-fertaric acid) were extracted and purified according to the method described by Vrhovsek (1998) [68]. *cis*-Resveratrol and *cis*-piceid were produced by photochemical isomerization of the *trans* forms, as described by Mattivi et al. (1995) [69]. Milli-Q water was used for the chromatography.

### 4.2. Samples

The fruits of the species *Spondias mombin* L. (taperebá) were collected in the southeastern part of the Federal District (15°54′16.50″ S and 47° 22′37.49″ W, at 855 m altitude), which is in the Cerrado biome, by Embrapa Cerrados (CPAC), Brazilian Agricultural Research Corporation (Embrapa), Federal District, Brasilia, Brazil. The fruits were harvested at the ripening stage and manually peeled, and the peel was used in further analysis. *Spondias mombin* peel was lyophilized and stored at −40 °C until the analyses. The protocol used for the extraction of the phenolic metabolites from the lyophilized sample was performed according to Vrhovsek et al. (2012) [70].

### 4.3. Elaboration of Taperebá Peel Extract

The extract was prepared according to Cabral et al. (2018) [26] from the peel of taperebá (*Spondias mombin* L.) using the ethanol extracting solution (ratio 1:2) and left in maceration for one week, under refrigeration and protected from light. Afterwards, the solution was filtered and stored in an amber flask and under refrigeration.

This extract was used to obtain microparticles (TMPs) and for the evaluation of total polyphenol content and antioxidant activity. For the stability study, the extract was lyophilized (Liotop Lyophilizer, model L101), and called lyophilized ethanol peel extract (LPE).

The preparation of taperebá peel extract to evaluate the profile of phenolic compounds by UPLC-MS/MS analysis was performed using the protocol of Theodoridis et al. (…) with adaptations described by Vrhovsek et al. (2012) [70]: 2 g of samples was extracted using 5 mL of water/methanol/chloroform (20:40:40) and vortexed for 1 min. At room temperature, the samples remained in an orbital shaker for 15 min. After that, samples were centrifuged at 1000× *g* and 4 °C for 10 min, and the upper phases were collected. Extraction was repeated by adding another 3 mL of water/methanol (1:2) to the pellet and shaking for 15 min. Then, the upper phases from the two extractions were blended, brought to 10 mL, and filtered through a 0.2 μm PTFE filter prior to analysis.

#### 4.3.1. Quantification of Total Phenolics and Evaluation of Antioxidant Activity

The phenolic extract quantification was performed using the Folin–Ciocalteau method [71]. Calculations were performed according to the standard curve of gallic acid (y = 0.0012x + 0.0273, R^2^ = 0.9979, LOD = 17.29 mg/mL, LOQ = 52.38 mg/mL), and the results were expressed in gallic acid equivalent (GAE) per 100 g of peel.

The evaluation of the in vitro antioxidant activity of the ethanol extract of taperebá peel was carried out by evaluating the capture of free radicals by DPPH [72] and ABTS [73]. Results were expressed as Trolox equivalent (TEAC) per g of peel and calculated from standard curves (DPPH: y = 0.0693x + 0.0406, R^2^ = 0.9993, LOD = 31.58 µmol/L, LOQ = 95.69 µmol/L; ABTS: y = 0.0726x + 1.6594, R^2^ = 0.9981, LOD = 53.39 µmol/L, LOQ = 161.78 µmol/L).

#### 4.3.2. Obtaining Chitosan Microparticles (MPs)

The medium molecular weight chitosan (Aldrich) MPs containing ethanolic extract of taperebá were obtained by spray drying (Labmaq, model MSD 1.0) according to the method of Cabral et al. (2018) [26] with adaptations. The extract was incorporated at a ratio of 4:1 (chitosan: dry extract), and the final solution in which 16 g of chitosan was used was dissolved in 800 mL of a 1% aqueous solution of acetic acid. Further, 4.7 g of dry extract (made by the dry weight of 200 mL of extract) was subjected to a spray dryer with: a flow rate of 7 mL/min, a pressurized atomizing nozzle of 1.0 mm in diameter, atomizing air at 30 L/min, hot drying air flow of 4.5 m^3^, inlet air temperature 140 °C and outlet air ~99 °C. The TMPs were then characterized and submitted to a stability study during storage.

##### Characterization of Chitosan Microparticles Containing Taperebá Peel Extract (TMP)

The quantification of phenolic compounds in TMPs was performed using the Folin–Ciocalteau method [71], with adaptations by CABRAL et al. (2018) [26]. Calculations were performed according to the standard curve, and the results were expressed as gallic acid equivalent (GAE) per g of microparticle.

The evaluation of yield and encapsulation efficiency of TMPs containing taperebá peel extract was performed according to the method of Gelfuso et al. (2011) [27] with the adaptations of Cabral et al. (2018) [26]. To characterize the morphology of the TMPs obtained, the sample was analyzed using a scanning electron microscope (SEM Jeol JSM-7000F), at a magnification of 1000 to 5000 times [9].

##### Microparticle Stability Study

The TMPs were submitted to a stability study regarding the total polyphenol content according to the Folin–Ciocalteau method, following the methodology carried out by Cabral et al. (2018) [26]. Stability was evaluated for a period of 60 days (days 0, 7, 14, 30 and 60) at three different temperatures: refrigerated (4 °C), ambient (~25 °C), and heat stress (~40 °C). The LPE was used as the control.

### 4.4. Profile of Phenolic Compounds—UPLC-MS/MS

Phenolic compounds were investigated by means of ultraperformance liquid chromatography coupled with mass spectrometry (UPLC-MS/MS). Liquid Chromatography: ULPC was performed on a Waters Acquity UPLC system (Milford, MA, USA). This system consists of a binary pump, an online vacuum degasser, an autosampler, and a column compartment. Phenolic compound separation was performed on a Waters Acquity HSS T3 column 1.8 μm, 100 mm × 2.1 mm (Milford, MA, USA), at 40 °C, according to Vrhovsek et al. (2012) [70]. An injection volume of 2 μL was used by both the standard solutions and the samples. The needle was rinsed with 600 μL of weak wash solution (water/methanol, 90:10) and 200 μL of strong wash solution (methanol/water, 90:10) after each injection. Samples were kept at 6 °C during the analysis.

Mass Spectrometry: Mass spectrometry detection was achieved on a Waters Xevo TQMS (Milford, MA, USA) instrument equipped with an electrospray (ESI) source, using the method previously described by Vrhovsek et al. (2012) [70]. The capillary voltage was 3.5 kV in positive mode and −2.5 kV in negative mode; the source was kept at 150 °C; the desolvation temperature was 500 °C; cone gas flow, 50 L/h; and desolvation gas flow, 800 L/h. A unit resolution was applied to each quadrupole. Flow injections of each individual metabolite were used to optimize the MRM conditions. For the majority of the metabolites, this was performed automatically by the Waters Intellistart software, whereas for some compounds the optimal cone voltages and collision energies were identified during collision-induced dissociation (CID) experiments and manually set. A dwell time of at least 25 ms was applied to each MRM transition.

Data Analysis. Data processing was performed using Waters MassLynx 4.1 and TargetLynx software (Waters, Milford, MA, USA). Data visualization and annotation of high-resolution spectra were performed using the R software suite (http://www.R-project.org, accessed on 3 March 2017), with specific use of the Gplot library for heat map graphics (Vrhovsek et al. (2012)) [70]. Standards were purchased or isolated as reported in the corresponding method used for the analysis of each class of compounds.

### 4.5. Statistical Analysis

All analyses were performed with two replications and in triplicate. A one-way ANOVA analysis (significance level 0.05) followed by Tukey’s test was performed using Statistica 13 software (Statsoft, Tulsa, OK, USA).

## 5. Conclusions

The results showed a significant content of total polyphenols in the peel of *Spondias mombin*, as well as high antioxidant power, demonstrating that it is an industrial residue with high functional value. Furthermore, it was possible to identify and quantify the major bioactives in the peel, highlighting the high content of ellagic acid and quercetin.

There was success in the microencapsulation process demonstrated by the SEM, in which photomicrographs showed the formation of TMPs, in addition to presenting satisfactory encapsulation efficiency and yield. The TMPs were able to increase the stability of the taperebá peel extract because during the 60 days of stability evaluation, in the heat stress condition, the TMPs preserved values of phenolic compounds that were almost twice as high, demonstrating greater retention at all evaluated temperatures. Thus, the results demonstrate that the microencapsulation process, from chitosan by spray drying, has proved to be a promising alternative way to increase the stability of phenolic compounds during storage, and thus enable their application.

To our knowledge, this study is, to date, the first to microencapsulate the ethanolic extract of taperebá peel (*Spondias mombin*), from the Cerrado biome of the midwest region of Brazil, using chitosan as an encapsulating polymer. Furthermore, it was the first to identify and quantify several phenolic compounds in the peel of this fruit, in addition to identifying and quantifying, for the first time, some compounds in the Spondias genus.

## Figures and Tables

**Table 1:** Quantification of metabolites from taperebá peel (µg/g) by UPLC-MS/MS.

Compound	Concentration (µg/g)
*Benzoic acid derivatives*	
2,5-Dihydroxybenzoic acid	0.102 ± 0.090
Gallic acid	21.994 ± 0.361
Vanillin	0.869 ± 0.080
Vanillic acid	0.006 ± 0.003
Methyl gallate	0.031 ± 0.010
Cinnamic acid	1.254 ± 0.040
Syringaldehyde	0.136 ± 0.053
Methyl anthranilate	0.003 ± 0.002
Ellagic acid	79.080 ± 3.272
*Coumarins*	
Daphnetin	0.377 ± 0.121
Esculin	4.016 ± 0.371
Scopoletin	0.382 ± 0.161
Fraxin	0.088 ± 0.030
*Phenylpropanoids*	
*p*-Coumaric acid	1.868 ± 0.211
Caffeic acid	0.239 ± 0.031
Ferulic acid	0.163 ± 0.025
Neochlorogenic acid	0.129 ± 0.011
Cryptochlorogenic acid	0.141 ± 0.012
Chlorogenic acid	10.236 ± 0.211
Sinapyl alcohol	1.142 ± 0.042
*trans*-Coutaric acid	0.036 ± 0.031
*Stilbenes*	
*trans*-Resveratrol	0.650 ± 0.022
*trans*-Piceid	3.577 ± 0.181
*cis*-Piceid	21.106 ± 1.330
*Dihydrochalcones*	
Phloretin	0.017 ± 0.004
Phloridzin	0.215 ± 0.041
Trilobatin	0.051 ± 0.011
*Flavones*	
Apigenin	0.035 ± 0.011
Sinensetin	1.453 ± 0.031
Luteolin	0.214 ± 0.011
Luteolin-7-*O*-Glucoside	1.185 ± 0.081
Hesperidin	0.129 ± 0.071
*Flavanones*	
Naringenin	1.037 ± 0.061
*Flavonols*	
Quercetin	66.402 ± 1.131
Quercetin-3-*O*-rhamnoside	0.305 ± 0.041
Quercetin-3-*O*-glucuronide	0.078 ± 0.021
Quercetin-3-Glc-Ara	0.065 ± 0.021
Rutin	1.326 ± 0.081
Kaempferol	0.374 ± 0.061
Kaempferol-3-*O*-glucoside	2.997 ± 0.091
Kaempferol-3-*O*-rutinoside	0.089 ± 0.011
Myricetin	8.137 ± 0.141
Syringetin	1.811 ± 0.051
Syringetin-3-*O*-glucoside	0.023 ± 0.011
Rhamnetin	0.150 ± 0.071
Isorhamnetin	0.769 ± 0.051
Isorhamnetin-3-*O*-glucoside	1.788 ± 0.081
*Hydroquinone derivative*	
Arbutin	1.279 ± 0.071

## Data Availability

Not applicable.

## Supplementary Materials

The following supporting information can be downloaded at: https://www.mdpi.com/article/10.3390/molecules27082382/s1, Table S1: List of the compounds included in the study, including CAS number, chemical formula, molecular weight and supplier.

## Sample Availability

Not available.
