# Supplementary material for: Phenolic Compound Profile by UPLC-MS/MS and Encapsulation with Chitosan of Spondias mombin L. Fruit Peel Extract from Cerrado Hotspot—Brazil
Source: Molecules. 2022 Apr 7;27(8):2382. doi: 10.3390/molecules27082382 (PMC9028924; doi:10.3390/molecules27082382)
Supplement: Supplementary file 1 [file molecules-27-02382-s001.zip › molecules-1631587-supplementary.pdf]

Table S1. List of the compounds included in the study, including CAS number, chemical formula, molecular weight and supplier.

Compound	CAS nº	METLIN ID	KEGG ID	Chemical formula	MW (Da)	Supplier
			Benzoic acid derivatives			
2,5-Dihydroxybenzoic acid	490-79-9	618	C00628	$C_7H_6O_4$	154.12	Aldrich
Gallic acid	149-91-7	3295	C01424	$C_7H_6O_5$	170.12	Aldrich
Vanillin	121-33-5	62927	C00755	$C_8H_8O_3$	152.14	Carlo Erba
Vanillic acid	121-34-6	5471	na	$C_8H_8O_4$	168.14	Carl Roth
Methyl gallate	99-24-1	44726	na	$C_8H_8O_5$	184.14	Fluka
Cinnamic acid	621-82-9	310	C10438	$C_9H_8O_2$	148.15	Aldrich
Syringaldehyde	134-96-3	na	na	$C_9H_{10}O_4$	182.17	Aldrich
Ellagic acid	476-66-4	3430	C10788	$C_{14}H_6O_8$	302.19	Fluka
			Coumarins			
Daphnetin	486-35-1	na	na	$C_9H_6O_4$	178.14	Biomedicals
Esculin	531-75-9	na	na	$C_{15}H_{16}O_9$	340.29	Carl Roth
Scopoletin	92-61-5	na	na	$C_{10}H_8O_4$	192.16	Fluka
Fraxin	524-30-1	na	na	$C_{16}H_{18}O_{10}$	370.31	Carl Roth
			Phenylpropanoids			
p-Coumaric acid	501-98-4	307	C00811	$C_9H_8O_3$	164.16	Sigma
Caffeic acid	331-39-5	3316	C01481	$C_9H_8O_4$	180.16	Sigma
Ferulic acid	1135-24-6	4156	C01494	$C_{10}H_{10}O_4$	194.18	Carl Roth
Neochlorogenic acid	906-33-2	na	na	$C_{16}H_{18}O_9$	354.31	TransMIT
Cryptochlorogenic acid	905-99-7	na	na	$C_{16}H_{18}O_9$	354.31	TransMIT
Chlorogenic acid	327-97-9	3498	C00852	$C_{16}H_{18}O_9$	354.31	TransMIT
Sinapyl alcohol	537-33-7	44805	C02325	$C_{11}H_{14}O_4$	210.23	Aldrich
trans-Coutaric acid	27174-07-8	na	na	$C_{13}H_{12}O_8$	296.23	Isolated
			Stilbenes			
trans-Resveratrol	501-36-0	6979	C03582	$C_{14}H_{12}O_3$	228.24	Isolated
trans-Piceid	27208-80-6	53240	C10275	$C_{20}H_{22}O_8$	390.38	Isolated
cis-Piceid	na	na	na	$C_{20}H_{22}O_8$	390.38	Isolated

			Dihydrochalcones			
Phloretin	60-82-2	3405	C00774	C15H14O5	274.26	TransMIT
Phloridzin	60-81-1	3535	C01604	C21H24O10	436.41	TransMIT
Trilobatin	4192-90-9	na	na	C21H24O10	532.45	Extrasynthese
			Flavones			
Apigenin	520-36-5	3397	C01477	C15H10O5	270.24	Carl Roth
Sinensetin	2306-27-6	49674	na	C20H20O7	372.36	Extrasynthese
Luteolin	491-70-3	3409	C01514	C15H10O6	286.24	Sigma
Luteolin-7-*O*-Glucoside	68321-11-9	na	C03951	C21H20O11	448.37	Sigma
Hesperidin	520-26-3	3678	C09755	C28H34O15	610.56	Sigma
			Flavanones			
Naringenin	480-41-1	3401	C00509	C15H12O5	272.25	Carl Roth
			Flavonols			
Quercetin	117-39-5	409	C00389	C15H10O7	302.24	Fluka
Quercetin-3-O-rhamnoside	522-12-3	43747	C01750	C21H20O11	448.38	Fluka
Quercetin-3-O-glucuronide	22688-79-5	na	na	C21H18O13	478.37	Extrasynthese
Rutin	153-18-4	3677	C05625	C27H30O16	610.52	Extrasynthese
Kaempferol	520-18-3	3410	C05903	C15H10O6	286.23	Carl Roth
Kaempferol-3-O-glucoside	480-10-4	64226	C12249	C21H20O11	448.37	Extrasynthese
Kaempferol-3-O-rutinoside	17650-84-9	50150	na	C27H30O15	594.53	Extrasynthese
Myricetin	529-44-2	3448	C10107	C15H10O8	318.23	Extrasynthese
Syringetin	4423-37-4	50914	C11620	C17H14O8	346.29	Extrasynthese
Syringetin-3-O-glucoside	40039-49-4	50904	na	C23H24O13	508.43	Extrasynthese
Rhamnetin	90-19-7	43977	C10176	C16H12O7	316.26	Extrasynthese
Isorhamnetin	480-19-3	3445	C10084	C16H12O7	316.26	Extrasynthese
Isorhamnetin-3-O-glucoside	5041-82-7	49007	na	C22H22O12	478.41	Extrasynthese

na= not available.
